# Editorial: The effects of mitochondrial dysfunction on the cell cycle

**DOI:** 10.3389/fcell.2023.1303834

**Published:** 2023-10-19

**Authors:** Danchen Wu, Lian Tian, Victoria Hoskin, Asish Dasgupta

**Affiliations:** ^1^ Department of Medicine, Queen’s University, Kingston, ON, Canada; ^2^ Strathclyde Institute of Pharmacy and Biomedical Sciences, University of Strathclyde, Glasgow, United Kingdom; ^3^ Division of Cancer Biology and Genetics, Department of Pathology and Molecular Medicine, Queen's Cancer Research Institute, Queen's University, Kingston, ON, Canada

**Keywords:** pulmonary arterial hypertension, cancer, mitochondrial fission, dynamin-related protein 1 (Drp1), cell cycle, diabetes, circular RNA (circRNA), cell proliferation

Hyperproliferative disorders, such as pulmonary arterial hypertension (PAH) and cancers, are characterized by excessive cell proliferation and resistance to apoptosis (Dasgupta et al., 2021). This “neoplastic phenotype” is due, at least in part, to acquired changes in mitochondrial metabolism. While perturbation of mitochondrial metabolism, notably a shift to aerobic glycolysis (*Warburg phenomenon*) contributes to the proliferation/apoptosis imbalance in cells from hyperproliferative disease origin, a newly recognized abnormality, namely, dysregulation of mitochondrial dynamics has been identified (Rehman et al., 2012). Mitochondria continuously join together (fusion) and divide (fission) thereby maintaining network quality control (Mao and Klionsky, 2013), mediating cell death (Tian et al., 2017) and regulating metabolism and the cell cycle (Chen et al., 2018). The major mediator of mitochondrial fission is dynamin-related protein 1 (Drp1); while fusion is meditated by mitofusin-1 and mitofusin-2 (Archer, 2013). Upon activation, Drp1 is recruited from the cytosol to the mitochondrial outer membrane (OMM) via interaction with its receptor proteins in a poorly understood multimerization reaction. In mammals, there are four proteins on the mitochondrial outer membrane that act as Drp1 receptors: mitochondrial fission 1 (Fis1), mitochondrial fission factor (Mff), mitochondrial dynamics proteins of 49 and 51 kDa (MiD49 and MiD51, respectively) (Atkins et al., 2016). Mitotic fission coordinates mitochondrial and nuclear division, ensuring equitable distribution of mitochondria between daughter cells. Mitotic fission occurs via a Drp1-dependent process. Several studies have shown that inhibition of mitotic fission triggers a cell cycle checkpoint and results in cell cycle arrest and apoptosis (Chen et al., 2018) both in cancers and in non-malignant, hyperproliferative diseases such as PAH (Marsboom et al., 2012). Thus, mitotic fission is an appealing therapeutic target. It has also been shown that Drp1 expression is upregulated in hyperproliferative diseases and Drp1 is postranslationally activated (Marsboom et al., 2012; Rehman et al., 2012; Tian et al., 2018; Abu-Hanna et al., 2023). Inhibition of Drp1 regresses cancer and PAH in animal models (Marsboom et al., 2012; Rehman et al., 2012). In this special edition of Frontiers in Cell and Developmental Biology, four journal articles on *The effects of mitochondrial dysfunction on the cell cycle* were published: Two original research articles, one review, and one mini-review. Three articles are relevant to pulmonary hypertension (PH), and the other is related to diabetes.

The perturbation of mitochondrial dynamics leading to disordered metabolism in PAH is thoroughly discussed in a comprehensive review by Breault et al. In this review article, the authors summarized the current knowledge on the causes and consequences of disordered mitochondrial function in PAH, focusing on aberrant mitochondrial metabolism, disruption of oxygen sensing, and abnormal mitochondrial dynamics in different cell and tissues in PAH.

A mini-review by Xiao et al. provided a comprehensive summary highlighting the mechanism of mitochondrial dynamics emphasizing the role of Drp1 in the pathogenesis of PAH. This mini-review article further summarized pharmacological inhibitors that are used to target Drp1. Drp1 is a large GTPase (Archer, 2013). Following activation, Drp1 is recruited to OMM where, at a site demarcated by the endoplasmic reticulum (Friedman et al., 2011), it assembles by multimerization with its receptor proteins by hydrolyzing GTP to form a macromolecular ring-like structure. This ring constricts and divides the mitochondria. Several approaches have been taken to inhibit Drp1’s ability to execute mitochondrial fission such as developing pharmacological inhibitors to inhibit Drp1’s GTPase activity (Mdivi1 and Drpitor1) (Cassidy-Stone et al., 2008; Wu et al., 2020). In addition to blocking Drp1’s GTPase activity, a competitive small peptide (P110) which blocks binding of Drp1 to its receptor protein Fis1 has been developed, thereby inhibiting Drp1’s recruitment to OMM (Qi et al., 2013). Inhibition of Drp1’s activity by mdivi-1/Drpitor1 or the Drp1-Fis1 interaction by P110 attenuated mitochondrial fission, cell proliferation, regressed cancer (Rehman et al., 2012; Wu et al., 2020) and improved right ventricular function during ischemia-reperfusion injury in experimental animal models (Tian et al., 2017). In addition, Fis1-specific inhibitor SS-31 is reported to regress PAH in a murine model (Lu et al., 2016).

In the research article entitled *Alterations in inflammatory cytokines and redox homeostasis in LPS-induced pancreatic beta-cell toxicity and mitochondrial stress: protection by azadirachtin*, the authors investigated the protective role of an anti-inflammatory, anticancer, and antioxidant phytochemical, azadirachtin (AZD) on the lipopolysaccharide (LPS)-induced nitro/oxidative stress in a rat insulin-secreting pancreatic beta cell line, Rin-5F (John and Raza). Using *in vitro* experiments, they discovered that the bacterial endotoxin LPS not only leads to nitro/oxidative stress but also triggers subsequent pro-inflammatory changes in cells, which is marked by translocation of nuclear factor kappa B (NF-κB) and release of inflammatory cytokines, such as tumor necrosis factor alpha (TNF-α) and interleukin 6 (IL-6). Furthermore, LPS also impaired mitochondrial function in Rin-5F cells. It reduced mitochondrial membrane potential, decreased electron transport chain (ETC) complex activities, decreased ATP production, and induced mitochondria-mediated apoptosis. AZD can successfully reverse these detrimental changes in pancreatic beta cells caused by LPS.

The pancreatic beta cell produces, stores, and releases insulin, a hormone that lowers blood glucose levels (Marchetti et al., 2017). Insulin plays a crucial role in glucose homeostasis. Beta cell dysfunction is a hallmark of metabolic syndrome (MetS) and the degree of β cell dysfunction correlates with the severity of MetS (Hudish et al., 2019). In the early stage of MetS, to compensate for the increased metabolic demand caused by changes in lifestyle, there is increased secretion of insulin in pancreatic β cells (Hudish et al., 2019). However, if the imbalance continues, β cell function will decompensate, eventually leading to a vicious loop and exacerbating MetS (Hudish et al., 2019).

Decompensated β cells in MetS and diabetes demonstrate many cellular pathological changes, including oxidative stress, inflammation, and mitochondrial dysfunction. These changes eventually lead to cell apoptosis. Increased plasma level of glucose, as seen in MetS and diabetes, induces glucose transportation across β cell membrane. As a result of increased glucose oxidative metabolism, the level of its byproduct, reactive oxygen species (ROS), is elevated. Mitochondria is the main organelle that produces ROS in β cells (Turrens, 2003). Excessive ROS exhausts the cytosolic antioxidants. In insulin-secreting cells, acute ROS attacks result in long-lasting mitochondrial inactivation, including partial loss of electron transport chain components (Li et al., 2009).

Altered mitochondrial morphology, especially fragmented and swollen mitochondrial networks are observed in diabetic animal models and β cells from type 2 diabetes (T2D) patients (Higa et al., 1999; Bindokas et al., 2003; Mizukami et al., 2008; Dlaskova et al., 2010). However, manipulating mitochondrial dynamics proteins does not always normalize β cell function (Supale et al., 2012). Since mitochondrial fission and fusion are closely related to mitophagy and calcium regulation, the involvement of mitochondrial dynamics in β cell function requires further investigation.

AZD is a chemical compound belonging to the limonoid group and an extract of fruit from the Neem tree. It acts as an antifeedant and growth disruptor to insects. The pharmaceutical use of AZD in medicine is less explored. However, preliminary studies with insects have shown that it has protective roles against oxidative stress (Zhang et al., 2018). Therefore, it may be a promising pharmacological agent for treating β cell oxidative dysfunction.

In the research article entitled *CircGSAP regulates the cell cycle of pulmonary microvascular endothelial cells via the miR-942-5p sponge in pulmonary hypertension,* Sun et al. explored the molecular mechanism for cell proliferation, apoptosis and cell cycle in pulmonary microvascular endothelial cells (PMECs). Non-coding RNAs are important molecular regulators of RNA activity and protein function and play important roles in cardiovascular, pulmonary, and muscle diseases (Bonnet et al., 2020). Circular RNAs (circRNA), a new type of non-coding RNAs, form covalent-closed continuous loops and can regulate cellular functions by acting as microRNA or protein inhibitors (“sponges”) (Kristensen et al., 2019; Wang et al., 2022). Many circRNAs have been found to be dysregulated in plasma or lung tissues in PH and the circRNAs are predicted to regulate the function of pulmonary arterial cells such as endothelial cell and smooth muscle cells (SMCs), resulting in pulmonary vascular remodeling in PH (Wang et al., 2022). Circular RNA gamma-secretase activating protein (circGSAP) has been found to decrease in lung tissues from idiopathic PAH patients (Yuan et al., 2021). This study demonstrates that circGSAP is downregulated in lung tissues from chronic obstructive pulmonary disease (COPD)-PH patients and in hypoxic PMECs; upregulated miR-942-5p and downregulated SMAD4 are also observed in hypoxic PMECs. The authors also showed that decreased circGSAP promotes cell proliferation, apoptosis resistance and G1/S transition and circGSAP plays such role through competitively binding miR-942-5p to module SMAD4. These results demonstrate the important role of circGSAP/miR-942-5p/SMAD4 axis in regulating PMECs under hypoxic condition. However, no study is presented to demonstrate the role of this axis in other cell types in the lungs such as pulmonary artery SMCs and *in vivo* preclinical animal models of PH. Although this study did not investigate the mitochondrial function, loss of SMAD4 has been demonstrated to decrease mitochondrial respiration and increase mitochondrial fission in pancreatic cancer cells (Ezrova et al., 2021). In addition, numerous studies have demonstrated mitochondria are critical component in regulating cell cycle, cell proliferation, apoptosis and many cell functions in both health and diseases (Ryan et al., 2015; Dasgupta et al., 2020; Wu et al., 2021). Future work is therefore needed to identify the role of mitochondria in the pathway in both *in vitro* and *in vivo* studies.
